# Supplementary material for: Analysis of land cover evolution within the built-up areas of provincial capital cities in northeastern China based on nighttime light data and Landsat data
Source: PLoS One. 2020 Oct 1;15(10):e0239371. doi: 10.1371/journal.pone.0239371 (PMC7529268; doi:10.1371/journal.pone.0239371)
Supplement: Attachment — Submitted filename: renamed_d063c.docx [file pone.0239371.s001.docx]

**Dear Editors,**

Thanks very much for the time and effort you spent in reviewing our manuscript entitled “**Analysis of land cover evolution within the built-up areas of provincial capital cities in northeastern China based on nighttime light data and Landsat data” (ID: PONE-D-20-18472).** We studied the comments carefully and revised the manuscript in accordance with the comments of the editor exactly. The following is a point-to-point response to the comments of the Editor.

**………………………………………………………………………………………**

**Comment 1:** 1. Thank you for providing information on Figure 4, 6, and 7.

We note for the images using Landsat data, attribution is required (https://landsat.visibleearth.nasa.gov/):

“Landsat imagery courtesy of NASA Goddard Space Flight Center and U.S. Geological Survey” or “USGS/NASA Landsat”

Before we proceed, please add the attribution above to the figure captions of all figures using the Landsat data.

**Response:** Thank the Editor very much for the constructive comments. In order to add the contribution of Landsat data, we refer to the website https://landsat.visibleearth.nasa.gov/. In addition, we found the same example in the paper published in PLOS ONE. The example paper is “Hong L, Huang Y, Peng S. Monitoring the trends of water-erosion desertification on the Yunnan-Guizhou Plateau, China from 1989 to 2016 using time-series Landsat images[J]. Plos One, 2020, 15(2): e0227498.” The statement form of the Landsat data contribution is shown below:

Figure1 The example

Based on the above reference, we have made the following modifications to the manuscript:

**(1) Fig 4. Advantages and disadvantages of the urban built-up area boundary extraction based on single source data.** (a) Built-up area extraction results of Shenyang in 2000 based on single source data; (b) Schematic of omissions in the extraction results with Landsat data; (c) Schematic of omissions in the extraction results with the DMSP/OLS data. (USGS/NASA Landsat).

**(2) Fig 7.** **Extraction Results of the urban built-up areas by the maximum likelihood method.** (USGS/NASA Landsat).

**(3) Fig 8. Extraction results of the urban built-up areas by the multisource boundary pixel fusion method.** (USGS/NASA Landsat).

**(4) Fig 9. Land cover class results.** (USGS/NASA Landsat).

**………………………………………………………………………………………**

We appreciate for the warm work of the Editors earnestly, and hope that the corrections will meet with approval. Once again, thank you very much for your thoughtful comments and suggestions. Should you have any questions, please contact us without hesitation. Thanks and best regards!

Yours Sincerely,

Lishuang SUN

September 5, 2020
